# COVID-19 related stigma among the general population in Iran

**DOI:** 10.1186/s12889-022-14039-2

**Published:** 2022-09-05

**Authors:** Masoomeh Faghankhani, Hossein Nourinia, Ali Ahmad Rafiei-Rad, Aliyeh Mahdavi Adeli, Mohammad Reza Javadi Yeganeh, Hamid Sharifi, Hamidreza Namazi, Shaghayegh Khosravifar, Alaleh Bahramian, Mahdi Fathimakvand, Elnaz Golalipour, Fatemeh Sadat Mirfazeli, Hamid Reza Baradaran, Graham Thornicroft, Amir Hossein Jalali Nadoushan

**Affiliations:** 1 Mental Health Research Center, School of Behavioral Sciences and Mental Health, Iran University of Medical Sciences, Mansouri Street, Niyayesh Street, Satarkhan Avenue, Tehran, 1445613111 Iran; 2 Academic Center for Education, Culture, and Research, Humanities and Social Studies Research Center, 47 Nazari Street, Abureyhan Street, Enghelab Avenue, Tehran, 141554364 Iran; 3 HIV/STI Surveillance Research Center, and WHO Collaborating Center for HIV Surveillance, Institute for Futures Studies in Health, Kerman University of Medical Sciences, Haft-Bagh Highway, Medical University Campus, 7616911320 Kerman, Iran; 4 School of Medicine, Iran University of Medical Sciences, Shahid Hemmat Highway, Next to Milad Tower, Tehran, 1449614535 Iran; 5 Department of Sociology, Faculty of Social Sciences, University of Tehran, Jalal Al-e Ahmad Highway, Tehran, 1411713118 Iran; 6 Department of Medical Ethics, Faculty of Medicine, Tehran University of Medical Sciences, PourSina Street, Qods Street, Tehran, 1417613151 Iran; 7 Medical Ethics and History of Medicine Research Center, Tehran University of Medical Sciences, PourSina Street, Qods Street, Tehran, 1417613151 Iran; 8 Department of Psychiatry, School of Medicine, Isfahan University of Medical Sciences, Hezar Jerib Street, Isfahan, 8174673461 Iran; 9 Department of Psychiatry, School of Behavioral Sciences and Mental Health, Iran University of Medical Sciences, Mansouri Street, Niyayesh Street, Satarkhan Avenue, Tehran, 1445613111 Iran; 10 CEO, Armandar Company, 16 Kambiz Street, West Zartosht Street, Apt. 7., Tehran, Iran; 11 School of Medicine, Shahid Beheshti University of Medical Sciences, Arabi Avenue, Daneshjoo Boulevard, Velenjak, Tehran, 1983963113 Iran; 12 Department of Epidemiology, School of Public Health, Iran University of Medical Sciences, Shahid Hemmat Highway, Next to Milad Tower, Tehran, 1449614535 Iran; 13 Ageing Clinical and Experimental Research Team, Institute of Applied Health Sciences, University of Aberdeen, Aberdeen, Scotland AB25 2ZD UK; 14 Centre for Global Mental Health and Centre for Implementation Science, Institute of Psychiatry, Psychology and Neuroscience, King’s College London, 16 De Crespigny Park, London, SE5 8AF UK

**Keywords:** Social stigma, Enacted stigma, COVID-19, Mental health, Pandemics, Urban population, Iran, Surveys and questionnaires, Cross-sectional studies

## Abstract

**Background:**

COVID-19 related stigma has been identified as a critical issue since the beginning of the pandemic. We developed a valid and reliable questionnaire to measure COVID-19 related enacted stigma, inflicted by the non-infected general population. We applied the questionnaire to measure COVID-19 related enacted stigma among Tehran citizens from 27 to 30 September 2020.

**Methods:**

A preliminary questionnaire with 18 items was developed. The total score ranged from 18 to 54; a higher score indicated a higher level of COVID-19 related stigma. An expert panel assessed the face and content validity. Of 1637 randomly recruited Tehran citizens without a history of COVID-19 infection, 1064 participants consented and were interviewed by trained interviewers by phone.

**Results:**

Item content validity index (I-CVI), Item content validity ratio (I-CVR), and Item face validity index (I-FVI) were higher than 0.78 for all 18 items. The content and face validity were established with a scale content validity index (S-CVI) of 0.90 and a scale face validity index (S-CVI) of 93.9%, respectively. Internal consistency of the questionnaire with 18 items was confirmed with Cronbach’s alpha of 0.625. Exploratory factor analysis revealed five latent variables, including “blaming”, “social discrimination”, “dishonor label”, “interpersonal contact”, and “retribution and requital attitude”. The median of the stigma score was 24 [25th percentile: 22, 75^the^ percentile: 28]. A large majority (86.8%) of participants reported a low level of stigma with a score below 31. None of the participants showed a high level of stigma with a score above 43. We found that the higher the educational level the lower the participant’s stigma score.

**Conclusion:**

We found a low level of stigmatizing thoughts and behavior among the non-infected general population in Tehran, which may be due to the social desirability effect, to the widespread nature of COVID-19, or to the adaptation to sociocultural diversity of the large city.

## Background

The Coronavirus disease 2019 (COVID-19) spread rapidly as far as the World Health Organization (WHO) officially declared a global pandemic on March 11, 2020 [1]. By January 2022, it has caused at least 5,502,856 death [2]. The ongoing COVID-19 pandemic has led to chaotic social interactions like COVID-19 related stigmatizing behavior and discrimination. COVID-19 related stigma targets patients, their families, and some communities [3, 4]. For example, incidents of stigmatization towards healthcare workers, COVID-19 patients, and survivors have been reported across the world [5–8]. As we know, health related stigma may deter people from adopting healthy behaviors like delay appropriate healthcare-seeking, which results in an increased psychological, social, economic, and physical burden of any disease [9–13]. It leads to detrimental effects on society’s health and optimal control of an infectious outbreak [14].

Our literature review revealed that COVID-19 related perceived stigma has been well discussed among healthcare workers; COVID-19 survivors; COVID-19 patients; people at high risk of contagion; and community populations like Asians who are U.S. residents, or students [6–8, 13, 15–24]. In contrary, the COVID-19 related enacted stigma was only investigated among healthcare workers, hospital visitors, and students, who are not representative of the general population [25–29]. Furthermore, the COVID-19 related enacted stigma from the non-infected general population’s perspective and their stigmatizing and discriminatory thoughts and behavior have not been satisfactorily addressed [15]. Moreover, most of the previous studies used a single item rather than a valid and reliable comprehensive scale to assess the discrimination or enacted stigma due to COVID-19 [15, 21, 30–34]. On the flip side, the few, who used the scale to measure stigma, had adapted the scale from stigma questionnaires pertaining to either similar infectious outbreaks or general physical/mental health issues [15, 35–37]. Therefore, a valid and reliable comprehensive scale to exclusively measure the COVID-19 related enacted stigma, imposed by the non-infected general population, was deemed necessary, especially in the Persian language.

Taken together, gap of knowledge on the perspectives of the non-infected general population about COVID-19 related enacted stigma, lack of a valid and reliable comprehensive scale to exclusively measure the COVID-19 related enacted stigma among the non-infected general population, and lack of a national study on COVID-19 related enacted stigma and importance of that made us determined to conduct this study. In this study, we developed, validated, and tested the reliability of a questionnaire to measure enacted stigma, inflicted by the non-infected general population. We also measured the COVID-19 related enacted stigma using our valid and reliable questionnaire in Tehran, Iran.

## Methods

### Setting and procedure

The institutional review board and ethics committee of Kerman University of Medical Sciences approved this study (IR.KMU.REC.1399.090). This study presented a multistep approach to develop, validate, and test the reliability of a questionnaire to measure COVID-19 related stigma, imposed by the non-infected general population, and measure the COVID-19 related stigma among the non-infected general population of Tehran from 27 to 30 September 2020.

### Questionnaire content and validity

We formed a pool of initial related and probable items through a comprehensive literature search using PubMed and Google Scholar. Our literature review yielded neither Persian nor English questionnaire of COVID-19 related stigma at that time. Hence, we included evidence about SARS and HIV/AIDS stigma in our search to extract the items of the SARS and HIV/AIDS related stigma questionnaires, and add those to our pool. We also reviewed COVID-19 related news using traditional media (i.e. newspapers, Television news) and COVID-19 related Hashtags using social media, including Instagram, Twitter, Facebook, and LinkedIn. Besides, we randomly discussed the topic with twenty individuals from the non-infected general population by phone.

Our team, composed of a sociologist, an epidemiologist, and two psychiatrists, suggested 18 items reflecting negative attitude towards COVID-19 disease (Items: 5, 6, 9, 16, 18), negative attitude towards the COVID-19 patients (Items: 1, 2, 3, 10, 11), enacted stigma in society (Items: 4, 12, 14, 15), and enacted stigma in interpersonal interactions (Items: 7, 8, 13, 17). The three-point scale, ranging from 1 to 3 while 1 means disagree, 2 means neither agree nor disagree and 3 means agree, was used to measure the participants’ agreement with each item (Table 1). The score on the questionnaire is computed by reversing responses (e.g., 1 = 3, 2 = 2, & 3 = 1) to the items 3, 4, 7, 9, 12, 14, 15, and 16, and then summing across all scale items. The total score ranges from 18 to 54. The higher score indicates the higher level of COVID-19 related stigma, inflicted by the non-infected general population. Based on a statistical classification, i.e. equality of distance between three classes of mild, moderate, and severe stigma score, we used the formula of the maximum score minus minimum score divided by three (54.18/3 = 12). Therefore, 12 is the distance between the classes. Therefore, a score ranging from 18 to 30 demonstrates a low level of stigma, 31 to 42 shows a moderate level of stigma, and 43 to 54 identifies a high level of stigma.

**Table 1:** Description of the COVID-19 related stigma questionnaire

Items	1%	2%	3%
1. COVID-19 patients are careless individuals with high-risk behaviors.	60.3	13.6	26.1
2. Contracting COVID-19 is the result of violating health regulations and is a clue to spot irresponsible violators.	44.5	12.7	42.8
3. It is not right to say that COVID-19 patients are lousy and filthy.	23.2	4.6	72.2
4. COVID-19 patients should not be excluded from society.	7.4	2.2	90.4
5. Contracting COVID-19 leads to embarrassment and disrepute in the patient.	82.5	4.5	13.0
6. COVID-19 stigma and disgrace will remain on the patients forever.	92.1	2.4	5.5
7. I do not have any issues to have close contact with recovered COVID-19 patients.	16.9	6.2	76.9
8. I prefer not to have even a recovered COVID-19 patient as my neighbor or colleague in my workspace.	67.0	7.0	26.0
9. COVID-19 is not divine retribution.	19.0	8.2	72.8
10. COVID-19 patients would like others to get infected too.	84.9	8.2	6.9
11. Fortunately, COVID-19 only affects the elderly and immunocompromised patients. #	53.8	2.4	6.9
12. COVID-19 patients’ right should be a priority.	2.5	4.6	92.9
13. The first COVID-19 patients in each city should be identified and penalized due to their role in spreading the disease.	69.2	8.9	21.9
14. COVID-19 patients should not be neglected.	9.0	1.7	89.3
15. An employer has no right to fire an employee infected with COVID-19.	8.1	3.4	88.5
16. A COVID-19 patient is never supposed to cause embarrassment and shame to his/her family.	10.0	2.1	87.9
17. COVID-19 stigma will be attached to the name of some affected cities forever.	88.0	4.5	7.5
18. Dying due to COVID-19 is the worst way to pass out.	57.4	9.1	33.5

Table 1 described the 18 items used in the COVID-19 related stigma questionnaire and the frequency of the participants’ agreement with each item, which was calculated in percent based on 1064 participants’ responses. Column 1 showed the rate of disagreement with each item, Column 2 indicated the frequency of neither agree nor disagree choice, and Column 3 displayed the rate of agreement with each item. #: Out of 1064 respondents, 393 respondents (36.9%) refused to answer question no. 14.

### Face, content, and construct validity

We recruited ten experts, who were affiliated to either Iran University of Medical Sciences or Kerman University of Medical Sciences, to the expert panel using an announcement. We accepted the volunteers on a rolling basis. An expert panel consisted of a sociologist, two emergency physicians, four psychiatrists, an epidemiologist, a specialist of medical ethics, and a physician-scientist. Their comments were applied to improve the questionnaires. Finally, they qualitatively validated the content, face, and construct of the preliminary draft. They also approved the questionnaire in terms of grammar, wording, item allocation, and scaling (Table 1).

Moreover, we measured the relevance, clarity and comprehension, simplicity, and essential nature of each item. The experts were asked to rate each item using a four-point ordinal rating scale to measure: relevance (1 = not relevant, 2 = somewhat relevant, 3 = quite relevant with minor revision and 4 = highly relevant); clarity and comprehension (1 = not clear and understandable, 2 = somewhat clear and understandable, 3 = quite clear and understandable with minor revision, and 4 = very clear and understandable); and simplicity (1 = not simple, 2 = somewhat simple, 3 = quite simple with minor revision, and 4 = very simple). The experts also rated each item using a three-point rating scale to measure essentiality (1 = not beneficial and not essential, 2 = beneficial but not essential, 3 = beneficial and essential).

Furthermore, the content validity index (CVI) was calculated using the relevancy measure. The content validity index of each item (I-CVI) was calculated as the number of experts who rated the item as highly relevant divided by the total number of experts. The item would be relevant if I-CVI ≥ 0.78. I-CVI ranges from 0 to 1. The value was closer to 1, the item would be more relevant. The content validity of the entire questionnaire tool, the scale content validity index (S-CVI), was considered as the average of the I-CVIs. The questionnaire as a tool was assumed as valid if S-CVI ≥ 0.90 [38, 39].

We used the essentiality measure to compute the content validity ratio of the item (I-CVR). I-CVR was calculated by Lawshe’s formula (CVR = [(E. (N / 2)) / (N / 2)]). In Lawshe’s formula, E indicated the number of panelists rating the item as “beneficial and essential”, and N indicated the total number of panelists. I-CVR ranges from .1 to 1. The value was closer to 1, the item would be considered more essential. I-CVR of at least 0.78 was assumed necessary to deem an item as an essential one. If the value was between 0.70 and 0.78, the item would need a revision, and if the value was below 0.70 the item would be eliminated [39].

To calculate the face validity index (FVI) for each item (I-FVI), ten raters were requested to independently provide a score for each item based on the clarity and comprehension scale mentioned above. The number of raters giving an item a clarity and comprehension rating of 3 or 4 was divided by the number of the raters for each item. The item would be clear and understandable if I-FVI ≥ 0.78. FVI for scale (S-FVI) was calculated by averaging the I-FVI scores for all the items on the scale. At least 83% of the experts must approve it to confirm the face validity of the questionnaires [40].

We used exploratory factor analysis (EFA) to establish construct validity and to categorize the items into the unobserved latent variables. The sample adequacy for factor analysis tested using Kaiser-Meyer-Olkin (KMO) measure and Bartlett’s test of Sphericity. We considered the KMO value over 0.5 and a significance level for the Bartlett’s test below 0.05 as a substantial correlation in the data [41]. The factor extraction method was Principal Axis Factoring (PAF) to compute covariance/correlation matrix. Then the combination of Kaiser criterion and scree plot examination () was used to select the factors to retain. Finally, Varimax with Kaiser normalization, an orthogonal factor rotation method, was used to minimize the number of variables.

### Reliability

Of 9,259,000 citizens of Tehran, a total of 1637 participants were recruited to the study via stratified random sampling. A software randomly generated a phone number with the city code of Tehran. Two third of the numbers were the mobile numbers and one-third of those were the landline numbers. We reach out to them using a phone call. A total of 1064 participants consented and were interviewed by 12 trained interviewers by phone (Response rate: 65%). Only 630 participants (59.2% of the consented participants) completely responded to all 18 questions of the scale. Data were stored in Excel then extracted to the SPSS file.

To assess the internal consistency of the questionnaire, we calculated Cronbach’s alpha using the returned questionnaire of 630 anonymous participants. Besides, corrected item-total score correlation coefficient and Cronbach’s alpha value were calculated for each item if the item was deleted. The value for item-total correlation above 0.2 was considered as good discrimination indicator [42]. However, we did not remove the items with item-total correlation coefficient above 0.15 because it is an exploratory study and our scale is going to measure a general and broad characteristic [43]. Cronbach’s alpha value above 0.6 was considered acceptable to establish internal consistency [44, 45].

The distribution of the continuous quantitative data was tested by the One-Sample Kolmogorov–Smirnov test. The mean and standard deviation were reported for all numerical data with normal distribution, otherwise median and interquartile range (IQR) were reported. All categorical variables were reported in frequency. Mann-Whitney test was used to compare the mean ranks of the stigma scores between two groups, and Kruskal. Wallis test was used to compare mean ranks of the stigma scores between more than two groups. The acceptable alpha error was considered as 0.05. IBM SPSS statistic 26 was used for statistical analysis.

## Results

### Demographic characteristics of participants

**Fig. 1:**
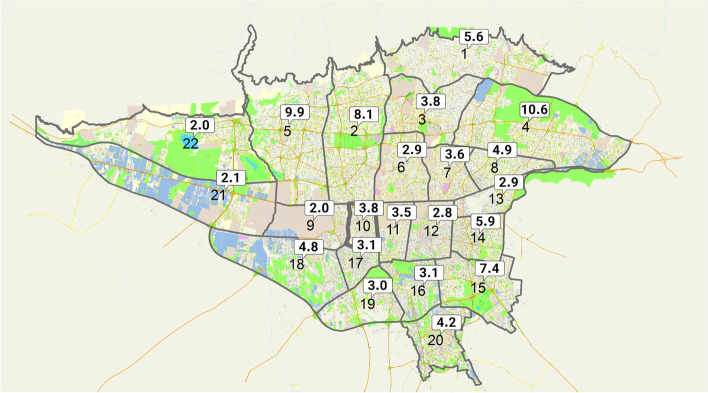
The distribution of participants across 22 districts in Tehran. It illustrated that how many percent of participants are from each of 22 districts of Tehran city on the map, which follows the same pattern of the distribution of the inhabitants across the city

The baseline characteristics of 1064 participants were summarized in Table 2. The median of participants’ age was 38 [25th percentile: 30, 75^the^ percentile: 52]. They were from various 22 districts in Tehran (Fig. 1). The participants reported that 71.8% of the people, with whom they interacted on a daily basis, took the COVID-19 pandemic as a serious issue. Similarly, 63.1% of participants described that their household strictly followed the COVID-19 self-isolation guideline. 55.7% of respondents had at least one COVID-19 patient among relatives, friends, or colleagues, while only 12.7% of them took care of a COVID-19 patient at the bedside. Besides, 41.2% of respondents were hit by the COVID-19 related death among their relatives, friends, or colleagues. The baseline characteristics of 630 participants, who completely responded to all 18 items of the questionnaire, followed the same pattern.

**Table 2:** Demographic characteristics of 1064 participants

Characteristics	Number of participants	Prevalence %
Gender		
Female	531	49.9%
Male	533	50.1%
Age Group in years		
< 30	296	27.8%
≥ 30 - < 40	305	28.7%
≥ 40 - < 50	189	17.7%
≥ 50 - < 60	141	13.3%
≥ 60	133	12.5%
Marital Status		
Married	728	68.6%
Never Married	287	27.0%
Loss of partner (Divorce or Death)	46	4.4%
Education		
College or higher degree	530	50.1%
Diploma of senior high school	333	31.5%
Junior high school or less	195	18.4%
Employment Status		
Employed	515	48.8%
Homemaker	325	30.8%
Retiree	101	9.6%
Student	63	6.0%
Out of work	50	4.8%

### Validity testing

The experts validated the content and face of the questionnaire. For each item, I-CVI, I-CVR, and I-FVI were higher than 0.78 (Table 3). For the questionnaire as a tool, S-CVI was 0.90, and S-FVI was 93.9%.

**Table 3:** Validity and reliability measures for the COVID-19 related stigma questionnaire

Items	I-CVI	I-CVR	I-FVI	Simplicity%	Corrected item-total score correlation	Alpha Cronbach if item deleted
1	0.88	0.80	0.95	95.0	0.331	0.595
2	0.90	0.78	0.92	95.0	0.233	0.612
3	0.90	0.80	0.97	95.0	0.217	0.614
4	0.90	1.00	1.00	100.0	0.207	0.614
5	1.00	1.00	1.00	100.0	0.247	0.609
6	1.00	1.00	0.97	97.5	0.312	0.605
7	0.90	0.95	0.95	97.5	0.161	0.621
8	0.89	0.80	0.87	87.5	0.325	0.595
9	0.78	0.80	0.92	97.0	0.211	0.614
10	1.00	1.00	1.00	100.0	0.256	0.609
11	0.78	0.78	0.87	95.0	0.215	0.613
12	0.89	0.80	0.87	90.0	0.067	0.627
13	0.90	0.80	0.95	92.5	0.255	0.607
14	0.88	0.78	0.87	97.0	0.160	0.619
15	0.88	1.00	0.92	92.5	0.187	0.617
16	0.89	0.80	0.95	100.0	0.231	0.611
17	0.90	0.78	0.87	92.5	0.281	0.606
18	1.00	0.78	1.00	100.0	0.236	0.611

Table 3 displayed the validity indexes, including content validity index of each item (I-CVI) or the relevancy, content validity ratio of each item (I-CVR) or the essentiality, face validity index of each item (I-FVI) or the clarity and comprehension, and simplicity for each item. It also represented the coherence between an item and the other items in the questionnaire using corrected item-total score correlation. In addition, it indicated the questionnaire’s Cronbach’s alpha reliability coefficient for internal consistency if the individual item is removed from the scale in the last column.

**Fig. 2:**
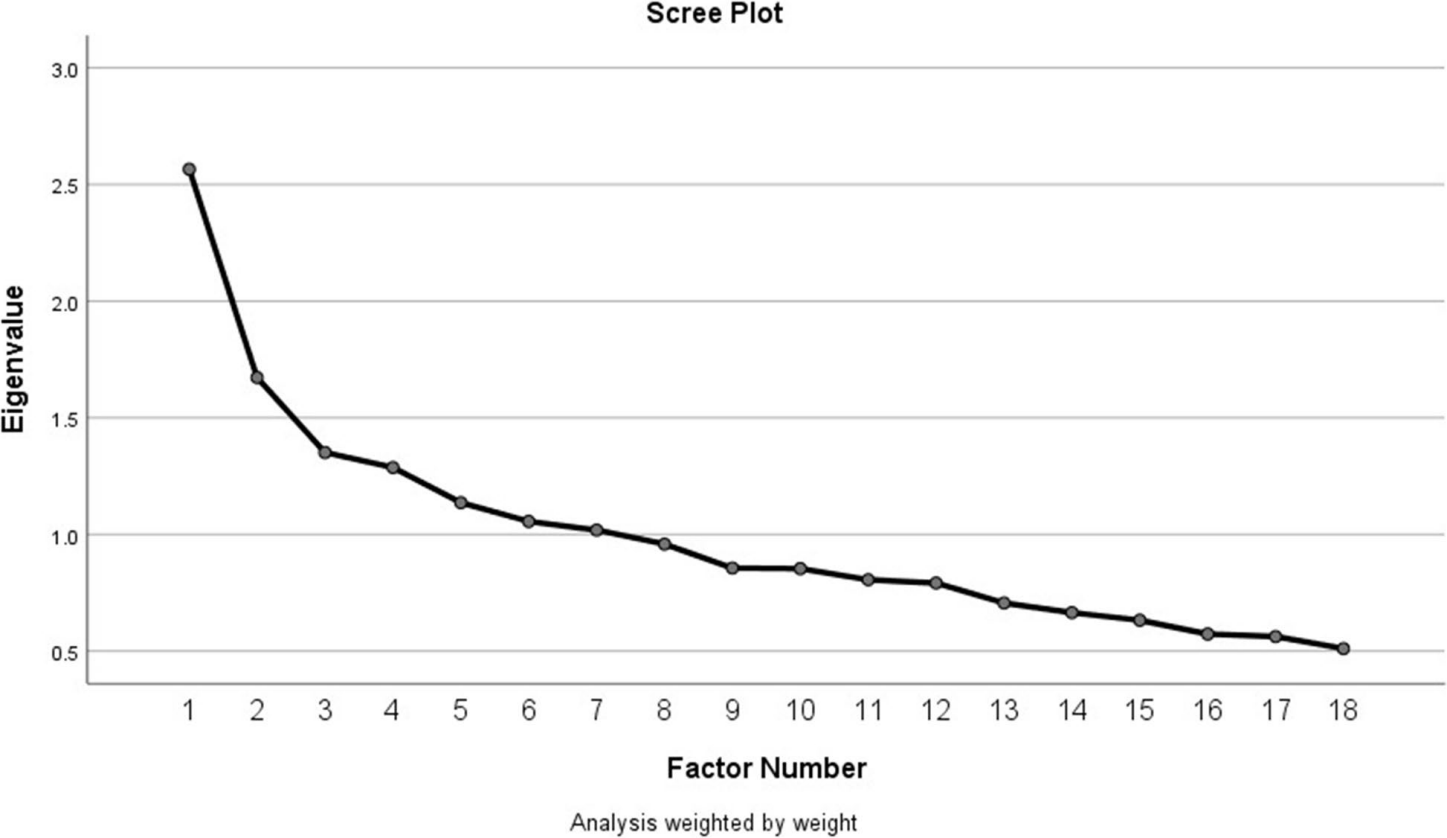
The scree plot of the exploratory factor analysis (EFA). The top screeplot illustrated the screeplot of preliminary EFA with 18 items and the bottom screeplot depicted the screeplot for final EFA with 11 items. Finally, we retained 11 factors with eigenvalue ≥0.9 and eliminate factors with eigenvalue <0.9 after identification of inflection line

**Fig. 3:**
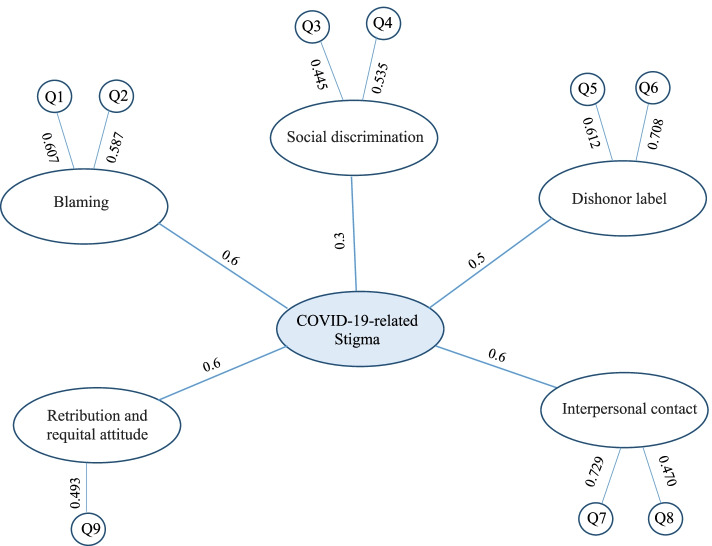
Structural model of factors consisting the COVID-19 related stigma imposed by non-infected general population. The exploratory factor analysis revealed five unobserved latent variables in the scale. Figure 3 depicted five unobserved latent domains (white ellipse) in the COVID-19 related stigma questionnaire. The questions, falling into each domain, were illustrated in white circles. The value on each arrow, connecting the white circles (item) to the white ellipses (latent factor), showed the correlation coefficient; however, the values on each arrow, connecting the white ellipses to the central ellipse (COVID-19 related stigma), represent the Cronbach Alpha of each latent variable to express the reliability of those latent factors

“The KMO overall measure of adequacy (MSA) for 18 items was 0.7, implying the adequate number of samples for factor analysis (Bartlett’s test of Sphericity; *P*<0.001, χ^2^:1005.47). Preliminary exploratory factor analysis with 18 items revealed seven factors. However, the correlation matrix depicted that seven items (Item 10 and 12.17) showed a low level of correlation (correlation coefficient ≤ 0.4) with their factors [46]. Furthermore, several numbers of those items showed a positive correlation with more than one factor, which implies the ambiguity of those items. Therefore, items 10 and 12.17 were deleted in the following exploratory factor analysis. The KMO overall measure of adequacy (MSA) for 11 items was 0.6, which implies that the sample must be cautiously taken adequate for factor analysis with 11 items (Bartlett’s test of Sphericity; *P*<0.001, χ^2^:567.05). Final exploratory factor analysis (followed the same method used in first EFA) with 11 items revealed five latent variables, including “blaming”, “social discrimination”, “dishonor label”, “interpersonal contact”, and “retribution and requital attitude”. Each factor had an eigenvalue ≥0.5 (Fig. 2). Two items (Item 11 and 18) did not fall in under any of the latent factor categories due to low level of correlation with other items (correlation coefficient ≤ 0.4) [46]. The four factors consisted of two items, and only “retribution and requital attitude” factor consisted of one item (Fig. 3). “Blaming” consisted of the following items “COVID-19 patients are careless individuals with high-risk behaviors” and “Contracting COVID-19 is the result of violating health regulations and is a clue to spot irresponsible violators”. “Social discrimination” consisted of the following items “It is not right to say that COVID-19 patients are lousy and filthy” and “COVID-19 patients should not be excluded from society”. “Dishonor label” consisted of the following items “Contracting COVID-19 leads to embarrassment and disrepute in the patient” and “COVID-19 stigma and disgrace will remain on the patients forever”. “Interpersonal contact” consisted of the following items “I do not have any issues to have close contact with recovered COVID-19 patients.” and “I prefer not to have even a recovered COVID-19 patient as my neighbor or colleague in my workspace”. Finally, “retribution and requital attitude” consisted of one item “COVID-19 is not divine retribution”. Cronbach alpha of five latent variables is 0.6, 0.3, 0.5, 0.6, 0.6, respectively.”

### Reliability testing

The internal consistency of the questionnaire with 18 items was confirmed by Cronbach’s alpha of 0.625. Item 12 was not removed; although, the corrected item-total correlation coefficient for that was too low, because the Cronbach’s alpha would not change significantly (Table 3). Since the internal consistency of the questionnaire with 11 items was low (Cronbach’s alpha: 0.54) compared to the 18.item version of the questionnaire, we retained all the items; however, items 10.18 fell out of five latent variables in the exploratory factor analysis due to low level of correlation.

### Covid-19 related stigma among the non-infected general population in Tehran

**Fig. 4:**
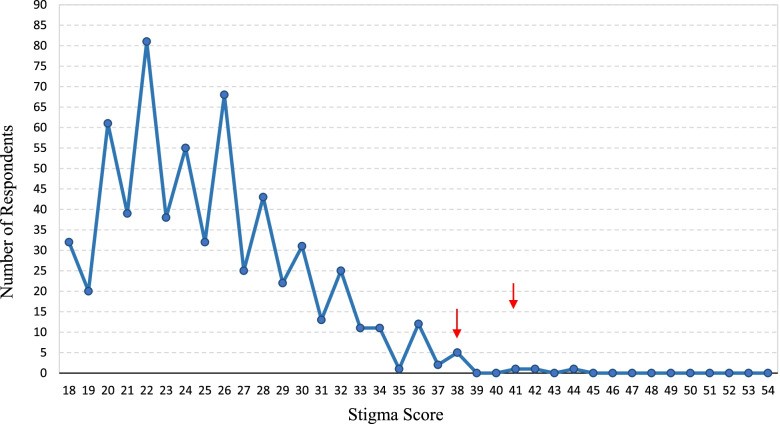
The frequency of each stigma score among 630 participants fully responded the questionnaire. It demonstrated the frequency of each stigma score among the respondents. X-axis represents the full range of the score from 18 to 54; Y-axis represents the number of respondents. Red arrows demonstrated no significant spike after score 38 and no change in the slope of the blue line above zero after score 44

The median of the stigma score was 24 [25th percentile: 22, 75^the^ percentile: 28] among 630 participants, who completely responded to all items. 86.8% of participants reported a low level of stigma with a score below 31. 13.2% of them demonstrated a moderate level of stigma, and none of the participants showed a high level of stigma with a score above 43 (Fig. 4). Subgroup analysis demonstrated that the mean rank of total stigma score was decreased with increase in the educational level [H (df: 2): 33.566; *P* < 0.001]. Similarly, mean rank of total stigma score was decreased among participants who took care of a COVID-19 patient at bedside and were exposed to at least one COVID-19 patient among relatives, friends, or colleagues [Mann-Whitney U (z: .3.9): 14912.0 and Mann-Whitney U (z: .2.8): 42394.0; *P* < 0.001 and P:0.005, respectively].

Further analysis showed that mean rank was increased among the participants with the lower level of education for “blaming”, “dishonor labeling”, and “retribution and requital attitude” factors [H (df: 2): 45.575, H (df: 2): 7.966, and H (df: 2): 9.810; *P* < 0.001, P:0.019, and P:0.007 respectively]. Further, mean rank was increased among participants who are a retiree, a student, or out of work for “blaming” factor [H (df: 4): 26.777; *P* < 0.001]. The highest mean rank was among participants with age group ≥60 and the lowest mean rank was among participants with age group ≥30 to < 40 compared to other age groups for “blaming” factor [H (df: 4): 33.009; *P* < 0.001]. Moreover, the mean rank was higher among homemakers and then students for “retribution and requital attitude” factor [H (df: 4): 20.659; *P* < 0.001]. The mean rank was also increased among women and never married group for “retribution and requital attitude” factor [Mann-Whitney U (z: .4.0): 41959.0 and Mann-Whitney U (z: .2.3): 33988.0; *P* < 0.001 and P:0.022, respectively]. In contrast, mean rank was decreased among participants who took care of a COVID-19 patient at bedside and were exposed to at least one COVID-19 patient among relatives, friends, or colleagues for “blaming” factor [Mann-Whitney U (z: .3.8): 20977.0 and Mann-Whitney U (z: .2.3): 45296.5; *P* < 0.001 and P:0.022, respectively] as well as “Interpersonal contact” factor [Mann-Whitney U (z: .4.3): 14944.0 and Mann-Whitney U (z: .3.1): 43275.5; *P* < 0.001 and P:0.002, respectively].

Out of 1050 responders answering item No. 2, 450 responders (42.8%) agreed to the stigmatizing statement “Contracting COVID-19 is the result of violating health regulations and is a clue to spot irresponsible violators.”, which is comparable to the number of responders (467 (44.5%)) disagreed to that. Furthermore, male participants reported a statistically significantly higher rate of agreement and a lower rate of disagreement to item No. 5, stating “Contracting COVID-19 leads to embarrassment and disrepute in the patient”, compared to the female participants [15.8% vs. 10.1 and 80% vs. 85%, respectively; P:0.019]. Besides, participants who had college or higher degree education reported a statistically significantly higher rate of agreement with item No. 5 compared to those who had a diploma of senior high school education or those who had junior high school education or less [14.2% vs. 11.2% vs. 12%, respectively; P:0.008]. Moreover, female participants showed a statistically significantly higher rate of agreement and a lower rate of disagreement to item No. 18 stating “Dying due to COVID-19 is the worst way to pass out.” compared to the male participants [38.7% vs. 28.4 and 51.4% vs. 63.3%, respectively; *P* < 0.001] (Table 1).

## Discussion

In this study, we developed a valid and reliable Farsi/Persian questionnaire to measure COVID-19 related stigma among the non-infected general population, with at least a junior high school education, in Iran. That scale comprised five latent factors, including “blaming”, “social discrimination”, “dishonor label”, “interpersonal contact”, and “retribution and requital attitude”. Cronbach alpha for social discrimination dimension and dishonor dimension were low due to the following reasons. The number of items in those dimensions are not sufficient to fully represent the factors. Besides, we had to substitute 5.point Likert scale with 3.point Likert scale to the measure of responses due to feasibility issues in collecting data. It dramatically reduced the reliability per se. We measured COVID-19 related stigma among the non-infected general population in Tehran, the capital of Iran. We found a low level of stigmatizing thoughts and behavior among the non-infected general population in Tehran, which may be due to the social desirability effect, widely spreading of COVID-19, or adaptation to the sociocultural diversity of the large city. Furthermore, our findings indicated that the educational level were weakly correlated with the participant’s stigma score. Therefore, the COVID-19 related stigma may not follow the same pattern in communities with either a higher rate of population with a lower level of education like low access or underserved areas of the country.

Moreover, thoughts and attitudes endorsing the “blaming” factor in the COVID19 related stigma scale were associated with a lower level of education, aging, and having the choice of skipping face-to-face social interaction in an occupational life such as retirees, out of work, or students versus employees. In contrast, bedside attendance; nursing; or having a COVID-19 patient among relatives, friends, or colleagues were associated with alleviation in blaming thoughts and attitude as well as avoidance of social interaction with COVID-19 survivors. Besides, the findings implied that the participants with lower education were inclined toward the “dishonor labeling” factor, which has been supported by the descriptive data showing the higher rate of agreement with the “Contracting COVID-19 leads to embarrassment and disrepute in the patient” statement among that group. Besides, Women, non-married participants, and participants with lower education expressed a stronger belief in the verdict stating that COVID-19 is a kind of divine retribution and requital. However, those correlations should be confirmed in specifically-designed future studies.

The descriptive data demonstrated an uncertainty on the “Contracting COVID-19 is the result of violating health regulations and is a clue to spot irresponsible violators.” statement among the participants, which might be due to that the responders assumed the question the two syllables with separated assumptions.

To the best of our knowledge, our study is the first one measuring COVID-19 related enacted stigma, imposed by the non-infected Iranian general population using a valid and reliable new scale that was exclusively designed for the COVID-19 pandemic. According to a systematic review about the prevalence of COVID-19 related stigma, by June 2021, 12 studies had investigated the prevalence of COVID-19 related stigma, including perceived or enacted stigma, among the general population in different countries: Egypt, Jordan, India, China (four studies), U.S.A. (three studies), Canada, Columbia, and the Philippines [15, 30–35, 38, 47–51]. Of those, three studies used a scale to measure the COVID-19 related enacted stigma, while other studies simply asked a single question or multiple ones about social discrimination due to COVID-19; stigma, associated with reporting travel history to the high-risk epidemic region; or stigmatizing nature of COVID-19. Of those three studies that used a scale, Cassiani-Miranda’s scale, from Columbia, is composed of 11 items [33]. Of these, seven items were adopted from the scale of stigma towards tuberculosis, developed by Upegui-Arango et al., and four items were added by authors [52, 53]. Cassiani-Miranda’s scale measures stigmatization and fear related to COVID-19. However, the homogeneity tests and the factorial analysis were not satisfactory. Therefore, it was used as multiple items rather than a scale [33]. The second one, Abuhammad’s scale, from Jordan, consists of eight items [35]. That was adapted from a questionnaire, originally developed by See et al., assessing the professional attitude of healthcare workers toward serving HIV/AIDS patients and drug users. The original scale consisted of four constructs: discrimination, acceptance of HIV/AIDS patients, acceptance of drug users, and fear [34]. Abuhammad’s scale is a valid and reliable questionnaire, which measures the stigma towards previously infected individuals or people exposed to COVID-19 such as healthcare workers. Although the target population of original and adapted scales, i.e. healthcare workers versus the general population, was not matched, the items in Abuhammad’s scale were rational, pragmatic, and relevant to the COVID-19 related stigma, enacted by the general population [35]. The third one, Taylor’s scale is a face-valid and reliable eight-item questionnaire, which was made for that study by authors. It measures the stigmatizing attitudes of the US and Canadian general population towards HCWs during the COVID-19 pandemic. The subject of the stigma in Taylor’s scale is only the healthcare workers rather than the whole COVID-19 issue, including the nature of the disease, patients, and survivors; although, the target population of Taylor’s scale was the general population. It was not adopted from previous scales, and it was valid and reliable [50]. Table 4 summarizes the characteristics of all 12 studies (Table 4).

**Table 4:** The characteristics of the studies investigated COVID-19 related stigma from general population’s perspectives

Study [Ref.]	Country; Population	Participants	Sample size; sampling method	Type of stigma	Prevalence of stigma	Measurement tool and Data collection method
Chen, 2020 [47]	China; Urban and rural	Adult residents of Hubei	1902; Unknown	Perceived	44.3%	Items: Perceived discrimination (whether they had encountered discrimination because of the COVID-19 pandemic). Online and Phone interview.
Wei, 2020 [48]	China; Urban and rural	Residents of 9 provinces with varying epidemic levels.	1344; Convenience	Perceived	57.4%	Items: Reporting travel history to high-risk epidemic region makes me feel stigma. Online.
Li, 2021 [30]	China; Urban and rural	Unvaccinated adult residents of 27 cities of 9 provinces	2377; Cluster Random	Enacted	62.3%	Items: Discrimination against recovered COVID-19 patients (6 questions with the content of face to face contact with recovered COVID-19 survivors). Face-to-face interview and online video interview.
Wang, 2021 [31]	China; Urban and rural	Adult residents of China	4191; Unknown	Enacted	62%	Items: Public discrimination against COVID-19 patients. Online.
Zhang, 2021 [36]	China; Unknown	Adult residents of Mainland China	1212; convenience	Enacted	31.8%^a^	Scale: Public Stigma of COVID-19 Scale. Online.
Zhao, 2021 [37]	China; Unknown	Adult residents of 26 provinces.	1920; Convenience	Enacted	@Max: 15.9% Min: 5.2%	Scale: COVID-19.related stigma toward individuals in high-risk areas, recovered patients with COVID-19, families of recovered patients with COVID-19, and frontline healthcare providers. Online.
Tee, 2020 [51]	Philippines; Urban and rural	Adult residents of Luzon Islands	1879; Snowball	Perceived	15.5%	Items: Feeling of being discriminated by other countries due to the outbreak of COVID-19. Online
Aqeel, 2020 [32]	India; Urban	Adult residents of Delhi.NCR	823; Systematic random	Enacted	73.3%	Items: COVID-19 infection has become a social stigma; Therefore, the patients are reluctant to disclose their symptoms at the early stage. Online.
Cassiani.Miranda, 2020 [33]	Columbia; Unknown	Columbian adults	1687; Convenience	Enacted	4.1%	Items^b^: Questionnaire on COVID-19 Stigma-Discrimination. Online.
Haddad, 2021 [54]	Lebanon; Urban and rural	Lebanese adults	405; Snowball	Enacted	62%^c^	Scale: Stigma discrimination scale. Online
Abuhammad, 2020 [35]	Jordan; Urban and rural	Jordanian adults	1655; Unknown	Enacted	64.8%^d^	Scale: Stigma toward COVID-19 infection scale. Online.
Abdelhafiz, 2020 [34]	Egypt; Urban and rural	Non-HCW Egyptian adults	559; Convenience	Enacted	22.7%	Items: Infection with the virus is associated with stigma. Online and face-to-face interview.
Taylor, 2020 [50]	U.S.A. and Canada; Unknown	Non-HCW adult residents of the U.S.A and Canada	3551; Random	Enacted	33.2%	Scale: HCW Stigmatization Survey; stigmatizing attitudes towards HCWs. Online.
Robinson, 2021 [49]	U.S.A.; Urban and rural	Adult residents of the U.S.A	5549; Random	Perceived	4.8%	Items: Four items adapted from the Perceived Everyday Experiences with Discrimination Scale, developed by Williams et al.^e^ Online.
Yu, 2020 [21]	U.S.A.; Unknown	Asian adult residents of 35 states of the U.S. A	235; Unknown Convenience	Perceived	7.6%	Items: Two items adapted from the Perceived Everyday Experiences with Discrimination Scale, developed by Williams et al.^e^ Online.
The current study	Iran; Urban	Adult residents of Tehran	630; Random	Enacted	13.2%^f^	Scale: COVID-19.related stigma questionnaire. Phone interview.

Table 4 described the 17 studies, including the current one, investigated perceived and enacted stigma from general population’s perspectives using either a single item or a scale. *Ref*. Reference, *HCW* Healthcare worker, *NCR* National Capital Region, *U.S.A.* United States of America.

^a^The mean: SD (Range of scale) of the stigma score was 2.68: 0.63 (1.5), indicating a mild to moderate level of stigma. @: Zhao et al. showed that 15.94, 14.84, 13.80, and 5.21% of participants endorsed stigma toward individuals in high-risk areas, recovered patients with COVID-19, families of recovered patients with COVID-19, and frontline healthcare providers, respectively. The mean: SD (Range of scale) of the stigma score for all subjects of stigma were between 2.03: 0.60 (1.5) and 2.38: 0.65 (1.5), indicating a mild to moderate level of stigma

^b^We considered the measurement tool of Cassiani-Miranda’s study an Item rather than a scale because they could not establish the reliability of their scale and results were reported based on each item.

^c^ The mean: SD (Range of scale) of the stigma score: 26.2: 5.4 (11.55), indication a mild to moderate level of stigma

^d^ The mean: SD (Range of scale) of the stigma score: 11.5: 1.3 (8.16), indicating a mild to moderate level of stigma

^e^Williams, D.R., Yu, Y., Jackson, J.S., & Anderson, N.B. (1997). Racial differences in physical and mental health: Socio-economic status, stress and discrimination. Journal of Health Psychology, 2, 335–351

^f^13.2% of participants reported a moderate level of stigma and 0% of them reported a severe level of stigma; The mean: SD (Range of scale) of the stigma score: 25.1: 4.7 (18.54), indicating a mild to moderate level of stigma

Furthermore, we found three newly-published studies that measured the COVID-19 related stigma and discrimination among the general population using a scale in China (two studies) and Lebanon [36, 37, 54]. Zhang et al. used the public stigma of the COVID-19 scale to measure stigmatizing attitudes towards COVID-19 among Chinese adults. Their scale, made up of 11 items, is valid and reliable [36]. That was adopted from the public stigma dimension of the stigma attached to the mental illness scale, originally developed by Mak et al. [55]. Zhao et al. also used a 14.item scale measuring the COVID-19 related stigma, including affective responses, cognitive responses, and behavioral intentions toward the following groups: individuals in high-risk areas, recovered patients with COVID-19, families of recovered patients with COVID-19, and frontline healthcare providers in China [37]. Their scale was adopted from Mak’s scale, originally measuring the level of stigma towards a disease among the general public [56]. However, Haddad et al. used a valid and reliable 11.item stigma discrimination scale developed by authors while being inspired by several previous scales measuring HIV/AIDS-related stigma (Table 4) [54, 57–59].

The highest rate of enacted stigma was reported from India, which was followed by Jordan China, Lebanon, the U.S.A/Canada, and Egypt regardless of the measuring tool was used (Items versus scale). In contrary to our findings, more than half of Lebanese and Jordanians demonstrated a moderate level of stigma and discrimination using a scale. However, it may be expected that due to geographical and cultural proximity, the prevalence of stigma in those countries should have been similar to ours. That may be because we did not include the rural population in our study; however, the participants of those studies were from both urban and rural populations. That may be also due to the difference between the sampling method of the studies: probability sampling versus non-probability sampling [35, 54]. Comparing Wang’s study with Zhang’s and Zhao’s, we noted that the prevalence of the stigma was dropped by half or more while the scale was used instead of an item to measure the COVID-19 related enacted stigma [31, 36, 37]. The prevalence of stigma in Zhao’s study, followed by Zhang’s, in China is the most similar one to our findings [36, 37]. The Jordanian, Chinese, and Lebanese general population indicated a mild to moderate level of stigma, which supported our findings among the Iranian general population [35–37, 54].

Moreover, previous studies mostly supported our findings of the correlates of COVID-19 related stigma among the general population. For instance, Zhang et al. reported that older age and lower level of education were significantly associated with higher stigma scores [36]. Haddad et al. also indicated that having a history of COVID-19 in the family and having direct contact with suspected or confirmed COVID-19 cases played the role of an ameliorating factor for the general population and reduced the stigma discrimination score [54]. Conversely, Zhao et al. detected that participants with master’s degrees or higher endorsed a higher level of stigma toward recovered patients. They also confirmed no significant differences among different ages regarding the level of stigma [37].

This study has several limitations. For instance, due to an inevitable factor of subjectivity when trying to assess stigma, it has a probability that respondents provide responses affected by social desirability factors, even though they were anonymous [60]. Moreover, non-response bias is a prevalent sampling bias in survey studies. People who refused to answer the questions or dropped out from a study may systematically differed from those who completely answer the stigma survey because we asked about an embarrassing information. We used some strategies to avoid sampling bias especially non-response bias. We defined the target population and sampling frame. We also match the sampling frame to the target population as much as possible. For example, we used a stratified random sampling method. We used a software randomly generated a phone number with the city code of Tehran. Two third of the numbers were the mobile numbers and one-third of those were the landline numbers. It followed the proportion of the communication method used by our population. Moreover, our statistic confirmed that the distribution of the participants followed the same pattern of the distribution of the inhabitants across 22 districts of the city. In addition, our survey was designed short enough by experts and trained staff to make data collection simple. We also used data collection by phone using a trained staff to develop a proper relationship with respondents to encourage them to cooperate, and make sure the respondents that any information given is completely confidential and anonymous [61]. Besides, 5.point Likert scale was preferred to use for rating each item in our questionnaire but we had to use 3.choice scale i.e. agree, neither agree nor disagree, and disagree rating scale due to improving understandability in oral communications. Since we administered our questionnaire by phone, we had to use an easily communicable, easily understandable, and a concise questionnaire to have an acceptable rate of participants’ attention and accurate answers. We believe that low Cronbach alpha for social discrimination dimension and dishonor dimension was due to these limitations. In addition, we included only urban population in our study due to executive limitation. Stigma against healthcare workers was not included in the context of this questionnaire. Moreover, since stigma is a dynamic concept and dependent on culture, social norms, rules, and conditions in each society, stigma varies by time and place. That scale was designed in the cultural and social context of Iran at the beginning of the pandemic. Although for the purpose of publication, two bilingual physicians translated these questionnaires to English, then two independent translators back-translated them to Persian. Finally, the translations were confirmed by questionnaire developers, we highly recommend to re-evaluate and adapting our questionnaire to other social and cultural contexts across the globe before use. Conversely, there are several strengths in our study. For instance, we developed a new valid and reliable scale to exclusively and specifically measure COVID-19 related stigma, imposed by the general population. Besides, we had a sample representative of urban population of Iran due to stratified random sampling method and using the residents of Tehran, as the most diverse city of Iran, for sampling frame. We also used both mobiles and landlines to overcome the limitations of random-digit dial telephone surveys. Moreover, we recruited participants from all districts of Tehran city based on the population of each district to match the sociodemographic characteristics of the sample with the those of population. Furthermore, we extracted the behaviors, thoughts, or attitudes that had made the COVID-19 related stigma in our community.

## Conclusion

Since COVID-19 related stigma and discrimination are a widespread and disturbing issue in a pandemic, it requires acknowledgment, screening, and prompt intervention to counteract it. Our questionnaire can play an essential role in screening the presence of the enacted stigma among non-infected general population, comprehending the different dimensions of that type of stigma from general population’s perspectives, and extracting the factors inspiring the prevention strategies. It is expected that the policymakers plan interventions and concerted actions to reduce and eradicate this health risk. We suggest that they target seniors, low. Educated communities, and female homemakers, students and out of work communities to enhance the impact of their interventions. We also suggest to focus on blaming and dishonoring mechanisms to better address the destigmatization in our society. It seems that our community needs a widespread and strong clarification about the lack of relationship between an infectious pandemic divine retribution and requital, and guilt.

## Data Availability

The datasets used and/or analyzed during the current study are available from the corresponding author on reasonable request.

## Abbreviations

COVID-19: Coronavirus disease of 2019
WHO: World health organization
SARS: Severe acute respiratory syndrome
HIV/AIDS: Human immunodeficiency virus/Acquired immunodeficiency syndrome
CVI: Content validity index
I-CVI: Content validity index of each item
S-CVI: Scale content validity index
CVR: Content validity ratio
I-CVR: Content validity ratio of each item
FVI: Face validity index
I-FVI: Face validity index for each item
S-FVI: Face validity index for scale
KMO: Kaiser-Meyer-Olkin
PAF: Principal axis factoring
SPSS: Statistical package for the social sciences
IQR: Interquartile range
SD: Standard deviation
No: Number
P: *P* value
vs: Versus
U.S.A.: United States of America
